# *Efficacy of Danlou Tablet* in Patients with Non-ST Elevation Acute Coronary Syndrome Undergoing Percutaneous Coronary Intervention: Results from a Multicentre, Placebo-Controlled, Randomized Trial

**DOI:** 10.1155/2016/7960503

**Published:** 2016-11-08

**Authors:** Lei Wang, Xujie Zhao, Shuai Mao, Shaonan Liu, Xinfeng Guo, Liheng Guo, Tinghai Du, Haiyu Yang, Fuhai Zhao, Keng Wu, Hongliang Cong, Yang Wu, Phillip C. Yang, Keji Chen, Minzhou Zhang

**Affiliations:** ^1^Department of Critical Care Medicine, 2nd Affiliated Hospital of Guangzhou University of Chinese Medicine, Guangzhou 510120, China; ^2^Chest Pain Center, Guangdong Provincial Hospital of Chinese Medicine, Guangzhou 510120, China; ^3^China-Australia International Research Centre for Chinese Medicine (CAIRC-CM), Guangdong Provincial Academy of Traditional Chinese Medicine, Guangzhou 510120, China; ^4^Department of Cardiovascular Medicine, First Affiliated Hospital of Henan College of TCM, Zhengzhou 450004, China; ^5^Department of Cardiovascular Medicine, Wuyi Hospital of TCM of Jiangmen City, Jiangmen 529000, China; ^6^Department of Cardiology, Xiyuan Hospital, Chinese Academy of Traditional Chinese Medicine, Beijing 100091, China; ^7^Department of Cardiology, Affiliated Hospital of Guangdong Medical College, Zhanjiang 524023, China; ^8^Department of Cardiology, Tianjin Chest Hospital, Tianjin 300051, China; ^9^Department of Cardiovascular Medicine, Oriental Hospital, Beijing University of Traditional Chinese Medicine, Beijing 100078, China; ^10^Department of Cardiovascular Medicine, Stanford University School of Medicine, Stanford, CA 94305, USA

## Abstract

This study seeks to investigate potential cardioprotection of Danlou Tablets in patients undergoing PCI with non-ST elevation acute coronary syndrome (NSTE-ACS). 219 patients with NSTE-ACS were randomised to Danlou Tablet pretreatment (*n* = 109) or placebo (*n* = 110). No patients received statins prior to PCI and all patients were given atorvastatin (10 mg/day) after procedure. The main endpoint was the composite incidence of major adverse cardiac events (MACEs) within 30 days after PCI. The proportion of patients with elevated levels of cTn I>5 × 99% of upper reference limit was significantly lower in the Danlou Tablet group at 8 h (22.0% versus 34.5%, *p* = 0.04) and 24 h (23.9% versus 38.2%, *p* = 0.02) after PCI. The 30-day MACEs occurred in 22.0% of the Danlou Tablet group and 33.6% in the placebo group (*p* = 0.06). The incidence of MACE at 90-day follow-up was significantly decreased in the Danlou Tablet group compared to the placebo group (23.9% versus 37.3%, *p* = 0.03). The difference between the groups at 90 days was the incidence of nonfatal myocardial infarction (22% versus 34.5%, *p* = 0.04). These findings might support that treatment with Danlou Tablet could reduce the incidence of periprocedural myocardial infarction in patients with ACS undergoing PCI.

## 1. Introduction

Over the past several decades, percutaneous coronary intervention (PCI) has emerged as the predominant therapeutic administration for ischemic heart disease. However, the high incidence of periprocedural myocardial infarction (PMI) following PCI severely impaired the benefits of coronary revascularization. It has been demonstrated that approximately 30% of patients undergoing PCI therapy developed PMI, which is significantly associated with bad long-term prognosis [1, 2].

Clinical trials have revealed the efficacy of high-dose statin treatment in significantly reducing the incidence of PMI among patients after coronary revascularization [3–5]. However, high-dose statin administration has been associated with severe side effects, including increased risk of new-onset diabetes, liver damage, rhabdomyolysis, and intracerebral haemorrhage [6–8]. Therefore, recent studies endeavoured to discover alternative natural agents that could reduce the incidence of myocardial necrosis after coronary intervention with low risk of side effects [9, 10].

**Figure 1:**
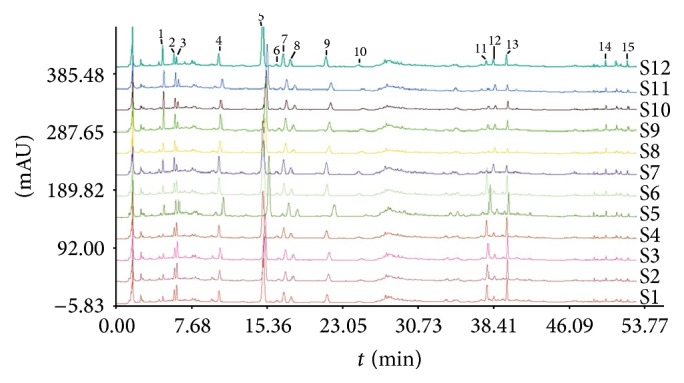
UPLC fingerprints of 15 batches of Danlou Tablets. 2: 5-hydroxymethyl furfural; 3: danshensu; 5: puerarin; 9: daidzin; 11: Sal B; 13: Sal A; and 15: tanshinone II A.

Over the past several years, there has been a surge of interest in the use of Chinese medicine for alleviating cardiovascular diseases, including angina pectoris, myocardial infarction, and chronic heart failure [11–13]. According to the theory of Traditional Chinese Medicine (TCM), the primary cause of coronary heart disease is intermingled phlegm and blood stasis. The* Danlou Tablet*, a patented Chinese medicine, has been approved by China Food and Drug Administration for patients with coronary heart disease and angina pectoris in 2005. The* Danlou Tablet* has been demonstrated to significantly alleviate phlegm and stasis mutual obstruction, decrease the serum level of inflammation molecular, and improve the quality of life in patients with unstable angina pectoris [14]. Furthermore, basic research has shown that it could decrease the area of myocardial ischemia and increase ion transport channel-related enzyme activities for the arrhythmia model rats induced by transient myocardial ischemia/reperfusion [15]. Ultraperformance liquid chromatography-tandem mass spectrometer (UPLC-MS/MS) was also used to analyse 15 quality-control markers of* Danlou Tablet*, and good consistency of the active markers was found among 12 different batches (Figure 1).

In the current study, we evaluated the hypothesis that* Danlou Tablet* treatment in patients with non-st-segment elevation acute coronary syndromes (NSTE-ACS) undergoing PCI would decrease the incidence of PMI and improve the clinical outcome by a multicentre, randomized, prospective, double-blind, placebo-controlled trial.

## 2. Methods

### 2.1. Study Design

The protocol of this clinical trial has been revealed in detail previously [16]. Briefly, the study followed the Declaration of Helsinki and was approved by Institutional Ethics Committee of Guangdong Provincial Hospital of Traditional Chinese Medicine and participating hospitals (Acknowledgments Section). Statin-naïve patients aged ≥18 years with non-ST-segment elevation ACS including unstable angina or non-ST-segment elevation myocardial infarction (NSTEMI) with selective coronary angioplasty to be undertaken within 72 h of admission were screened. Patients with acute ST-segment elevation myocardial infarction (STEMI), cardiac shock, severe heart failure (left ventricular ejection fraction <30%), or hepatic dysfunction with elevated alanine aminotransferase and aspartate aminotransferase serum levels for any reason were excluded from this study. Written informed consent was obtained from each participating patient.

### 2.2. Interventions

Eligible patients were randomized to either the experimental group or the control group. Patients in the experimental group received the treatment of* Danlou Tablet* (4.5 g per day for 48 hours before PCI and a further 4.5 g per day for 90 days after PCI). Patients in the control group received the administration of placebo. All enrolled patients received standard management care in accordance with the Guidelines for the Management of Patients with Unstable Angina/NSTEMI [17], including anti-platelet agents, anticoagulation agents, lipid-lowering agents, antiventricular remodelling, or antihypertensive therapy depending on the conditions of patients, irrespective of the randomization assignment. In particular, atorvastatin served as the unique lipid-lowering agent in the study, administrated with the moderate-intensity dose of 10 mg/day before and after PCI without any loading doses for the purpose of minimising the effect of statin on the results.

Enrolled patients were assessed at 48 h before PCI, 8 h and 24 h after PCI, and 30 days and 90 days after PCI. Assessments included physical examination, vital signs, quality-of-life measurement, electrocardiogram (ECG), and echocardiography. Furthermore, blood samples were collected for laboratory tests, including cardiac biomarkers (troponin I and creatine kinase-myocardial band [CK-MB]), lipids, biochemistry, haematology, C-reactive protein (CRP), and urinalysis.

### 2.3. Randomization and Blinding

Participants were randomly assigned into two groups. Randomization was stratified by centre with permuted block size. The randomized sequence was generated by computer with SAS 9.2 software (SAS institute Inc., Cary, USA) and saved in China-Australia International Research Centre for Chinese Medicine (CAIRC-CM), Guangdong Academy of Chinese Medical Sciences. Allocation of treatments was distributed by sealed, opaque, and numbered envelopes. Practitioners and participants were unaware of their assignment. One statistician who generated the blinding code was aware of the drug allocation. Participants, physicians, outcome assessors, and other statisticians and practitioners, however, remained blind to treatment assignments before the results were revealed. The placebo was prepared and packed by a pharmaceutical company (Jilin Connell Pharmaceutical Co. Ltd., China). The placebo was designed similarly to* Danlou Tablet* in shape, size, and taste. Each participant was provided with a bottle of water labelled with their unique number according to randomization.

### 2.4. Outcomes

The primary endpoints of this trial were the incidence of death, nonfatal myocardial infarction, target vessel revascularization (bypass surgery or repeat PCI), and rehospitalization due to acute cardiovascular events (severe angina or heart failure) within 30 days of the procedure. Secondary endpoints included the incidence of major adverse cardiovascular events (MACEs) within 90 days of PCI, the proportion of patients with elevated biomarkers of myocardial injury (troponin I) at 8 h and 24 h after PCI, and the proportion of patients with elevated CRP level at these time points. Nonfatal myocardial infarctions included spontaneous myocardial infarction, PMI, and myocardial infarctions related to stent thrombosis. PMI was defined as a postprocedural increase of cardiac troponin I (cTn I) values more than 5 × 99th percentile of the upper reference limit (URL) or a rise of cTn I values >20% if baseline values were elevated. Spontaneous myocardial infarctions were considered to be related to ischemia due to plaque erosion and/or rupture, fissuring, or dissection [18].

Furthermore, the safety and tolerability of experimental drugs were evaluated by the incidence, severity, and relationship to treatment of adverse events (AEs), including clinically significant changes in vital signs, physical examination findings, and laboratory measurements.

### 2.5. Sample Size Estimation

The sample size was calculated based on the reduction of incidence of MACE by a two-sided test with level size of 5% and a power of 80% chance of detecting a difference. A previous trial revealed the incidence of MACE as 17% in patients treated with low-dose statin therapy within 30 days after PCI [3]. Assuming a MACE incidence of 5% in the experimental group, it was calculated that a minimum of 99 patients would be required for each group. Additionally, we estimated that up to 10% of initial participants may withdraw from the trial (PASS 11.0 software, NCSS, Utah, USA). We thus determined that 232 eligible cases would be required.

### 2.6. Statistical Analysis

The trial database was blindly reviewed before the data were locked and unblended by the independent data collection centre, China-Australia International Research Centre for Chinese Medicine. Data from all participants who underwent randomization were analysed based on the intention-to-treat (ITT) principle. A per-protocol (PP) analysis was performed to test the robustness of the trial results. Baseline clinical and demographic characteristics were expressed as mean ± SD, median, or interquartile range (IQR). For comparisons, two samples were compared by* t-*test for normally distributed values; otherwise the Mann–Whitney* U* test was used. Proportions were analysed using the Chi-square test or the exact test method. Kaplan-Meier plots of cumulative incidence freedom of overall MACEs were constructed from the 30-day and 90-day follow-up. For all analyses, a value of *p* < 0.05 (two-tailed) was considered statistically significant. All calculations were conducted using SPSS software version 18.0 (IBM Inc., New York, USA).

## 3. Results

### 3.1. Patient Population

**Figure 2:**
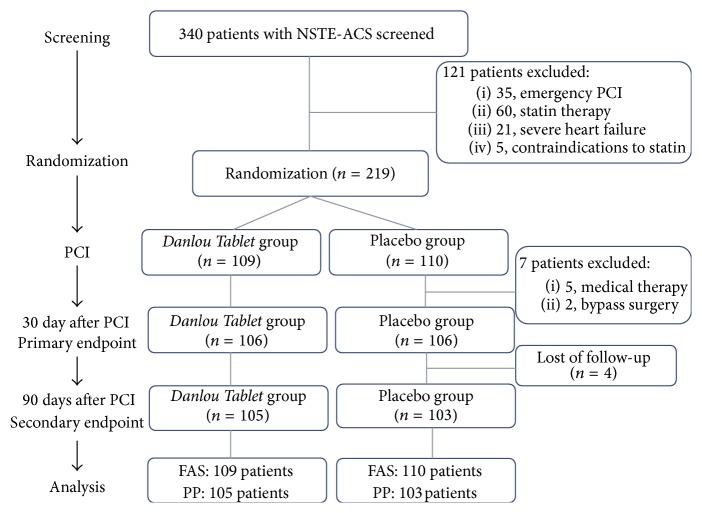
Flow diagram of Danlou Tablet for patients with ACS undergoing PCI. FAS, full-analysis set; NSTE-ACS, non-ST-segment elevation acute coronary syndrome; PCI, percutaneous coronary intervention; and PP, per protocol.

Between November 25, 2012, and March 23, 2014, 340 patients were screened: among them, 60 were excluded because of previous or current treatment with statins; 35 were excluded because they required an emergency PCI approach; 21 were excluded because of severe heart failure with ejection fraction <30%; and 5 were excluded because of contraindications to statin treatment (e.g., hepatic dysfunction or serious adverse reaction) (Figure 2). Eligible patients (*n* = 219) received the study assignment drug (*Danlou Tablet* or placebo) before PCI. Procedural success was obtained in all patients; 4 patients (2 in each group) had no-reflow or slow-flow phenomenon, which was significantly alleviated following intracoronary administration of platelet glycoprotein IIb/IIIa inhibitors (*tirofiban*). The last subject completed follow-up in June, 2014. Data entry was completed by November, 2014.

Demographic and clinical features of patients in the* Danlou Tablet* group and placebo group are indicated in Table 1. Patient characteristics were not significantly different in age, gender, cardiovascular risk factors, concomitant diseases, clinical presentation, cardiac function, blood creatinine levels, and medical therapy at the time of intervention. In comparing the main procedural features treatments in both the* Danlou Tablet* and placebo groups, there were statistically significant differences in the stent length (indicated in Table 2), but there were no statistically significant differences in coronary anatomy, multivessel lesion type, procedural characteristics of intervention, number of stents per patient, or the diameter of implanted stents (*p* > 0.05).

### 3.2. Cardiovascular Events

**Figure 3:**
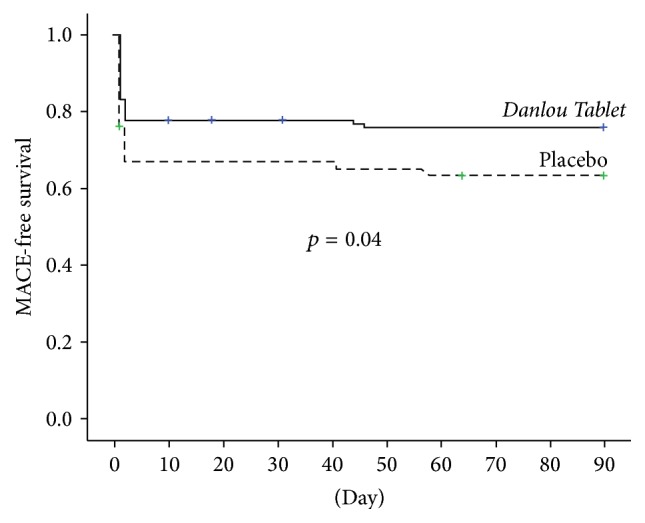
Survival curves. Kaplan-Meier curves of 90-day major adverse cardiac event- (MACE-) free survival in the 2 arms. MACE, major adverse cardiac event (death, nonfatal myocardial infarction, target vessel revascularisation, and rehospitalization due to acute cardiovascular events).

**Figure 4:**
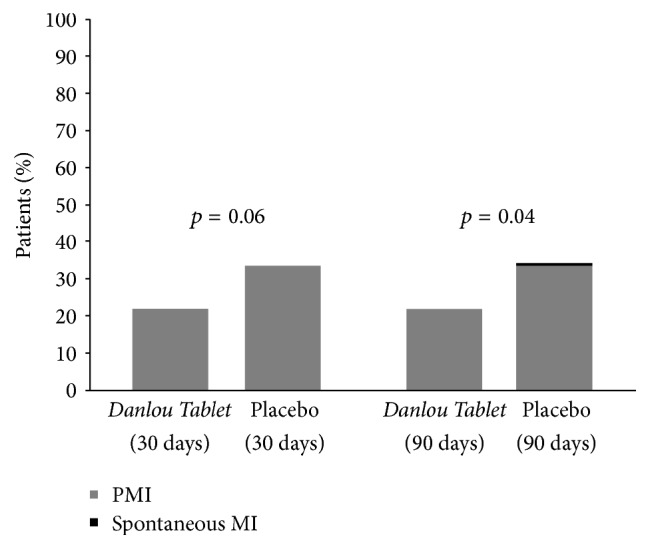
Non-fatal MI. ^*∗*^PMI: periprocedure myocardial infarction.

The primary endpoint was assessed at 30 days after coronary revascularization (Table 3). There was no statistically significant difference in the incidence of MACE in the* Danlou Tablet* group (22.0% of patients [24 of 109]) in the* Danlou Tablet* group compared to the placebo group (33.6% of patients [37 of 110]) (*p* = 0.06). At 90 days after PCI, however, the incidence of MACE in the* Danlou Tablet group* was significantly lower than in the placebo group (23.9% versus 37.3%, *p* = 0.03, Table 4). Furthermore, Kaplan-Meier curves showed that MACE-free survival rates at the 90-day follow-up were significantly greater in* Danlou Tablet* group than in the placebo group (*p* = 0.04, Figure 3). The lower rate of MACE at 90 days in the* Danlou Tablet* group was mostly driven by a reduced incidence of nonfatal myocardial infarction (22% versus 34.5%, *p* = 0.04). The vast majority of nonfatal myocardial infarction cases occurred within 24 hours after PCI and were defined as PMIs (Figure 4). One patient (0.9%) in the placebo group died due to probable stent thrombosis complicated with cardiac shock. In two patients (1.8%) in the* Danlou Tablet* group and five patients (4.5%) in the placebo group, in-stent restenosis occurred, and coronary revascularization had to be repeated. There was no statistically significant difference in the incidences of target vessel revascularization between the treated and placebo groups (*p* = 0.45).

### 3.3. Biomarker Results

**Figure 5:**
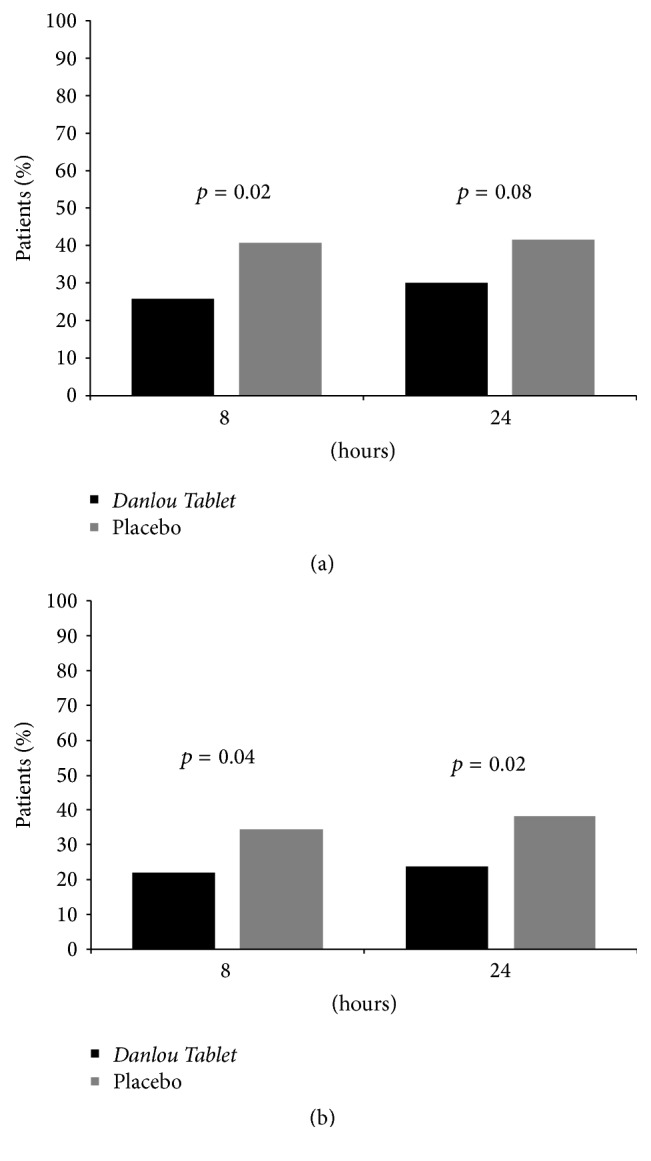
Cardiac Marker Elevations. (a) TnI >3 × 99th percentile of the upper reference limit (URL) at 8 and 24 hours after PCI in the Danlou Tablet group and in the placebo group. (b) TnI >5 × 99th percentile of the URL at 8 and 24 hours after PCI in the Danlou Tablet group and in the placebo group.

Before the procedure, there were no significant differences in the percentage of patients with abnormal cTn I elevation between two group (27.5% versus 30.0%, *p* = 0.69, Table 1). After the procedure, 8 and 24 hr post-PCI cTn I>3 × 99th percentile of URL occurred more frequently in the placebo arm than in the* Danlou Tablet* group (25.7% versus 40.9%, *p* = 0.02, and 30.3% versus 41.8%, *p* = 0.08) (Figure 5(a)). Moreover, the proportion of patients with elevated levels of cTn I>5 × 99th percentile of URL was significantly lower in the* Danlou Tablet* group at 8 h (22.0% versus 35.4%, *p* = 0.04) and 24 h after PCI (23.9% versus 38.2%, *p* = 0.02) (Figure 5(b)). Analyses on the population with elevated CRP levels showed no statistically significant differences between the* Danlou Tablet* group and the placebo group at 8 h (37.1% versus 32.0%, *p* = 0.44) and 24 h (47.6% versus 42.7%, *p* = 0.48) after PCI.

### 3.4. Safety Analysis

Only two patients in* Danlou Tablet group* had minor gastrointestinal adverse events (nausea and diarrhea), which were relieved after two days of suspension in the management of the experimental drug. Meanwhile, there were no significant ECG or physical examination findings or changes in laboratory parameters potentially associated with experimental drugs.

## 4. Discussion

It has been reported that a large number of patients undergoing PCI might suffer from myocardial injuries arising from the procedure itself, which is significant enough to serve as a negative prognostic factor [19]. Although previous studies have shown that pretreatment with high-dose statin is associated with reduced incidence of PMI in patients with acute coronary events [3, 4], still more studies did not demonstrate similar results [20, 21]. Particularly, a trial in which statin-naïve Korean and Chinese patients with NSTE-ACS received additional loading high-dose atorvastatin before PCI showed that there were no benefits compared with usual post-PCI atorvastatin treatment [21]. Moreover, recent trials revealed that there were no expected benefits of high-intensity statin treatments in East Asian patients compared with moderate- or low-intensity statin treatments [22, 23]. The reason why there were the different benefits of high-dose statin between Asian and Caucasians population could be attributed to the race difference of statin pharmacokinetics. Therefore, strong evidence-based natural agents to improve East Asian patient management and reduce the incidence of PMI are critical to address this challenge.

The present study was a multicentre, randomized controlled trial conducted in a statin-naïve Chinese ACS population undergoing selective PCI. Herein, we compare a group in which* Danlou Tablet* was administered for 2 days before elective PCI following a further treatment with the control group who received placebo based on usual care including moderate-intensity statin treatment (atorvastatin, 10 mg/day). Our study demonstrated that the proportion of patients with elevated levels of troponin I was significantly lower in the* Danlou Tablet* group at 8 h and 24 h, suggesting that* Danlou Tablet* pretreatment was associated with a significantly lower occurrence of PMI (Figure 5(b)). There was a tendency towards a lower occurrence of major cardiac events 30 days after PCI in patients with ACS undergoing selective interventional therapy (22.0% versus 33.6%, *p* = 0.06). The MACE-free Kaplan-Meier curves showed a significantly better event-free survival at 90 days in the* Danlou Tablet* arm. This change may be attributed to a significant reduction of nonfatal myocardial infarctions. The incidence of PMI in our trial was higher than that in the ARMYDAR-ACS [4] or ROMA trials [5]. The different diagnostic criteria of PMI may have led to this result; in the latter trials, PMI was diagnosed by the increase of creatine kinase-MB instead of cTn I.

The predominant mechanisms of PMI are complicated, including vulnerable plaque disruption [24], ischemia/reperfusion injury [25], oxidative stress, platelet activation [26], and inflammatory cytokines activation [27] induced by balloon pressure inflation or stent implantation during the procedure. The* Danlou Tablet* is a potent herbal compound mainly consisting of* Salvia, Ligusticum chuanxiong Hort, Trichosanthes kirilowii, *and* Allium macrostemon*. Chen et al. [28] report that* Danlou Tablet* may accelerate blood circulation and eliminate intravascular phlegm, which have critical roles in managing the ischemic disease based on the Traditional Chinese Medicine (TCM) theory. An increasing number of studies have been conducted to elucidate the cardioprotective effects of* Danlou Tablet*. Liu et al. investigated the efficacy of* Danlou Tablet* in improving cardiac function in miniature swine with experimental coronary disease and found that the* Danlou Tablet* treatment significantly improved cardiac function and histopathology and downregulated malondialdehyde (MDA) and superoxide dismutase (SOD) serum levels [29]. Guo et al. examined the effects of* Danlou Tablet* on arrhythmia model rats induced by transient myocardial ischemia/reperfusion (I/R) and revealed that* Danlou Tablet* inhibited the incidence of fatal and nonfatal ventricular fibrillation. The reduced frequency and duration of ventricular tachycardia might be related to lowering the degree of myocardial ischemia and increasing ion transport channel-related enzyme activities (Na^+^-K^+^ATPase and CaATPase) [15]. Similarly, our recent studies also suggested that administration of* Danlou Tablet* could alleviate the severity of risk-region myocardial ischemia and reperfusion arrhythmia* in vivo* [30]. Moreover, recent studies revealed that puerarin, the main component of* Danlou Tablet* by UPLC-MS/MS analysis (Figure 1), could significantly protect cardiomyocytes from anoxia/reoxygenation injury* in vitro* [31, 32].

In addition, endothelial injury induced by balloon or stent inflating is another key contributor for PMI [33]. Yang et al. found that the original superfine* Danlou Tablet* improved endothelial function in rats with arterial endothelial injury by downregulating the cardiac chymase signal pathway and chymase-mediated angiotensin II production [34]. Hong's study suggested that* Danlou Tablet* may protect vascular endothelium and improve formation of new capillaries on the edges of the ischemic myocardial zones, thereby contributing to the ultimate reduction of the infarct size and subsequent alleviation of the progressive heart failure [35]. Furthermore, another possible mechanism underlying the beneficial effects of* Danlou Tablet* may involve the alleviation of inflammatory reactions at the atherosclerotic plaque [28]. A clinical trial by Wang et al. showed the treatment of* Danlou Tablet *could alleviate inflammatory activation by reducing the serum level of hs-CRP, soluble CD40, and interleukin-6 [14]. However, our study demonstrated that there was no significant difference in the serum level of hs-CRP between two arms. Consequently, the anti-inflammation effects of this patented Chinese herb need to be further explored by more studies* in vitro* and* in vivo*.

Therefore, the benefits of* Danlou Tablet* in reducing PMI could be explained by the reported multiple mechanisms by which the treatment or its components inhibit ischemia reperfusion injury, decrease oxidative stress responses, and improve endothelial dysfunction and microvessel coronary circulation. Pre-PCI treatment of* Danlou Tablet *in NSTE-ACS patients may consequently translate into clinical positive outcomes.

## 5. Limitations

This study has some limitations. Firstly, the figures we used to estimate the sample size were not consistent with the actual results, which led to relatively lower power than expected. Secondly, biochemical measurements (e.g., CRP and cTn I) were not performed in the laboratory, which may have decreased the reliability of results. Furthermore, this trial was undertaken in China; whether the effects of the trial drugs would be similar in other ethnic groups is unknown.

## 6. Conclusion

In conclusion, the present trial implied that treatment with* Danlou Tablet* based on the low-dose of statin could reduce the incidence and magnitude of PMI and subsequently improve clinical outcomes in patients receiving elective revascularization with unstable angina and non-ST-segment elevation acute coronary syndromes. If confirmed by larger additional randomized studies, these findings may support the indication of “upstream” administration of this alternative natural agent in patients with acute coronary syndromes treated with an early invasive strategy.

## Figures and Tables

**Table 1:** Main demographic and clinical features of the two groups.

Variable	Danlou Tablet group (*n* = 109)	Placebo group (*n* = 110)	*χ* ^2^ (*Z*)	*p* value
Age, yr	62.89 ± 9.23	63.89 ± 10.03	0.04	0.44
Men, number (%)	72 (66.1)	74 (67.3)	0.04	0.85
Body weight, kg	66.91 ± 10.14	66.65 ± 9.54	−0.6	0.55
Height, cm	166.41 ± 6.85	166.00 ± 8.17	−0.23	0.82
Family history of coronary disease, number (%)	18 (16.5)	10 (9.1)	2.71	0.10
Concomitant diseases, number (%)				
Previous coronary heart disease	41 (37.6)	47 (42.7)	0.60	0.44
Previous coronary intervention	9 (8.3)	17 (15.5)	2.71	0.10
Systemic hypertension	51 (46.8)	63 (57.3)	2.41	0.12
Diabetes mellitus	21 (19.3)	19 (17.3)	0.15	0.70
Dyslipidemia	38 (34.9)	38 (34.5)	0.002	0.96
Heart failure	1 (0.9)	4 (3.6)		0.37^*∗*
Arrhythmia	4 (3.7)	10 (9.1)	2.69	0.10
Stroke	7 (6.4)	8 (7.3)	0.06	0.80
Current smoker, number (%)	47 (43.1)	44 (40.0)	0.22	0.64
Cardiac Marker Elevation, number (%)	30 (27.5%)	33 (30.0%)	0.16	0.69
Clinical pattern, number (%)				
Unstable angina	88 (80.7)	87 (79.1)	0.09	0.76
NSTEMI	21 (19.3)	23 (20.9)		
Cardiac function, number (%)				
Level I	41 (37.6)	41 (37.3)	0.03	0.99
Level II	53 (48.6)	53 (48.2)		
Level III	15 (13.8)	16 (14.5)		

Values are given as number of patients (%) or mean ± SD. NSTEMI, non-ST-segment elevation myocardial infarction; PCI, percutaneous coronary intervention; ACE, angiotensin-converting enzyme; and ARB, angiotensin II receptor blocker. ^*∗*^ *p* value is from Fisher's exact test.

**Table 2:** Angiographic and procedural features of the two groups.

Variable	Danlou Tablet group (*n* = 109)	Placebo group (*n* = 110)	*χ* ^2^ (*Z*)	*p* value
Multivessel coronary artery disease, number (%)	62 (56.9)	63 (57.3)	0.00	0.95
Vessel treated, number (%)				
Left main	2 (1.8)	2 (1.8)		1.00^*∗*^
Left anterior descending	73 (67.0)	65 (59.1)	1.46	0.23
Left circumflex	26 (23.9)	34 (30.9)	1.37	0.24
Right coronary artery	35 (32.1)	30 (27.3)	0.61	0.43
Restenotic lesions, number (%)	1 (0.9)	1 (0.9)		1.00
Multivessel intervention, number (%)	24 (22.0)	18 (16.4)	1.13	0.29
Type of intervention, number (%)				
Balloon only	1 (0.9)	1 (0.9)		1.00^*∗*^
Stent	108 (99.1)	109 (99.1)		1.00^*∗*^
Number of stents per patient	1.49 ± 0.70	1.41 ± 0.67	−0.89	0.37
Stent diameter, mm	2.94 ± 0.43	2.92 ± 0.43	−0.87	0.39
Total stent length, mm	20.52 ± 7.01	22.37 ± 7.46	−2.34	0.02

Values are given as number of patients (%) or mean ± SD. ^*∗*^ *p* value is from Fisher's exact test.

**Table 3:** MACE at 30 days after PCI in the Danlou Tablet and placebo groups.

	Incidence		Treatment difference (%)	
	Danlou Tablet group	Placebo group	Difference^a^ of incidence (95% CI)	*p* value
Full-analysis set	*N* = 109	*N* = 110		
Total MACE, *n* (%)	24 (22.0)	37 (33.6)	1.8 (1.0, 3.3)	0.06^c^
Cardiac death, *n* (%)	0	0		
Nonfatal MI, *n* (%)	24 (22.0)	37 (33.6)	1.8 (1.0, 3.3)	0.06^c^
Target vessel revascularization, *n* (%)	0	1 (0.9)		1.0^d^
Rehospitalization due to CVE^b^, *n* (%)	0	0		
Per-protocol analysis set	*N* = 107	*N* = 109		
Total MACE, *n* (%)	24 (22.4)	36 (33.0)	1.7 (0.9, 3.1)	0.08^c^
Cardiac death, *n* (%)	0 (0)	0 (0)		
Nonfatal myocardial infarction, *n* (%)	24 (22.4)	36 (33.0)	1.7 (0.9, 3.1)	0.08^c^
Target vessel revascularization, *n* (%)	0	1 (0.9)		1.0^d^
Rehospitalization due to CVE^b^, *n* (%)	0	0		

CI, confidence interval; CVE, cardiovascular events; MACE, major adverse cardiac event; and MI, myocardial infarction.

^a^Difference of incidence = Danlou Tablet − placebo.

^b^Cardiovascular events included severe angina or heart failure (NYHF *⩾* IV).

^c^ *p* value is from the continuity-adjusted Chi-square test.

^d^ *p* value is from Fisher's exact test.

**Table 4:** MACE at 90 days after PCI in the Danlou Tablet and placebo groups.

	Incidence		Treatment difference (%)	
	Danlou Tablet group	Placebo group	Difference of incidence (95% CI)	*p* value
Full-analysis set	*N* = 109	*N* = 110		
Total MACE, *n* (%)	26 (23.9)	41 (37.3)	1.9 (1.1, 3.4)	0.03^c^
Cardiac death, *n* (%)	0	1 (0.9)		1.00^d^
Nonfatal MI, *n* (%)	24 (22.0)	38 (34.5)	1.9 (1.0, 3.4)	0.04^c^
Target vessel revascularization, *n* (%)	2 (1.8)	5 (4.5)	2.5 (0.5, 13.4)	0.45^d^
Rehospitalization due to CVE^b^, *n* (%)	0	0		
Per-protocol analysis set	*N* = 105	*N* = 103		
Total MACE, *n* (%)	25 (23.8)	35 (34.0)	1.6 (0.9, 3.0)	0.11^c^
Cardiac death, *n* (%)	0	1 (1.0)		0.50^d^
Nonfatal MI, *n* (%)	23 (21.9)	32 (31.1)	1.6 (0.9, 3.0)	0.13^c^
Target vessel revascularization, *n* (%)	2 (1.9)	5 (4.9)	2.6 (0.5, 13.9)	0.28^d^
Rehospitalization due to CVE^b^, *n* (%)	0	0		

CI, confidence interval; CVE, cardiovascular events; MACE, major adverse cardiac event; and MI, myocardial infarction.

^a^Difference of incidence = Danlou Tablet − placebo.

^b^Cardiovascular events included severe angina or heart failure (NYHF *⩾* IV).

^c^ *p* value is from the continuity-adjusted Chi-square test.

^d^ *p* value is from Fisher's exact test.

## Abbreviations

ACS: Acute coronary syndrome
CHD: Coronary heart disease
CK-MB: Creatine kinase isoenzyme MB
CMR: Cardiac magnetic resonance
CRP: C-reactive protein
cTn I: Cardiac troponin I
DSMB: Data and Safety Monitoring Board
MACE: Major adverse cardiac event
NYHA: New York Heart Association
NSTE-ACS: Non-ST elevation acute coronary syndrome
PMI: Periprocedural myocardial injury
PCI: Percutaneous coronary intervention.
